# Taking Embodiment Seriously in Ethics and Political Philosophy

**DOI:** 10.1007/s10790-023-09952-7

**Published:** 2023-07-21

**Authors:** Joseph T. F. Roberts

**Affiliations:** University of Birmingham, Birmingham, UK

## Introduction

It is a common refrain in a certain brand of critical theorising that both liberal political philosophy and ethical theory pay insufficient attention to the fact that people are embodied. Martha Fineman, for example, argues that ‘our current system has been built upon myths of autonomy and independence and thus fails to reflect the vulnerable as well as dependent nature of the human condition.’^1^ Fineman suggests that this failure to take account of people’s embodied vulnerability leads to an impoverished account of equality which focuses on formal antidiscrimination provisions, rendering invisible other underlying social inequalities.^2^ This, in turn, makes these forms of inequality harder to address. Taking our nature as embodied vulnerable beings seriously, on the other hand, allows for a deeper understanding of equality, one that ‘brings institutions - not only individual actions - under scrutiny, re-directing our attention to their role in providing assets in ways that may unfairly privilege certain persons or groups’.^3^ Rachael Tilman argues similarly that forms of ethical argument that rely on abstraction and universalisation, as liberalism is said to do, ‘inevitably eliminate embodied, particular features of objects of knowledge in order to render them universal or substitutable.’^4^

As these examples show, the charge against liberal political and ethical theories tends to be directed at the conception of the self on which they are supposedly based. Liberalism is said to presuppose a disembodied conception of the self as a rational moral agent. The focus here is on notions of independence, autonomy, and self-sufficiency. This view of the self, it is argued, abstracts away from people’s particular embodiment, ignoring the ‘actual lived experience and the human condition’.^5^ In the words of Rosalyn Diprose:

In focusing on moral principles and moral judgments the assumption is that individuals are present as self-transparent, isolated, rational minds and that embodied differences between individuals are inconsequential.^6^

Rawls’ use of the veil of ignorance in the original position is a frequent target for this critique,^7^ mainly due to it being one of the most prevalent contractualist liberal theories in analytic philosophy. As the contracting parties are forced to abstract away from the particulars of their bodily existence when deciding principles of justice, Rawls is said to presuppose a disembodied view of the self. It is often claimed that considerations surrounding gender, disability status, or race are glossed over on Rawls’ view.^8^ This conception of the self as disembodied is sometimes traced back to Descartes’ distinction between the *res cogitans* (mind) and the *res extensa* (matter). Cartesian Dualism, like Rawls’ use of the veil of ignorance, is also said to abstract away from our embodied existence by conceiving of the mind and self as somehow separable.^9^

The critics who level these charges at liberalism (and the disembodied view of the self it is said to presuppose) are a varied group. They include feminist scholars such as Elizabeth Kingdom,^10^ Rosalyn Diprose,^11^ and Sherlyn Hamilton;^12^ communitarian political philosophers such as Michael Sandel,^13^ Charles Taylor,^14^ and Alasdair MacIntyre;^15^ phenomenologists such as Drew Leder,^16^ Fredrik Sveneaus,^17^ and S. Kay Toombs;^18^ disability scholars such as Eva Feder Kittay,^19^ Margrit Shildrick,^20^ and Jackie Leach Scully;^21^ Foucauldian theorists such as Ian Burkitt,^22^ Luna Dolezal,^23^ and Cressida Heyes;^24^ and vulnerability theorists and care ethicists such as Eva Feder Kittay,^25^ Martha Fineman,^26^ and Margaret Urban Walker.^27^

The critiques these different groups advance are varied. Feminist scholars advance the critique that disembodied views of the self are inherently patriarchal given the cultural association between femininity and the body and maleness and the mind.^28^ Communitarians tend to focus on how we are embedded in social practices which influence our values.^29^ Disability theorists emphasise how a disembodied view of the self as autonomous implicitly or explicitly excludes disabled people and obscures their experiences.^30^ Foucauldian theorists stress, amongst other things, how disciplinary power alters bodies.^31^ Phenomenologists highlight the importance of the interconnection of body and mind in constructing our subjectivity.^32^ Care ethicists and vulnerability theorists cast doubt on the primacy of independence and underscore the importance of dependency relationships in ethical theorising.^33^

Despite their differences, these theorists all have one thing in common. They converge on the idea that what is wrong with the disembodied view of the self is that it neglects the influence our bodies have on our lives. They are united in the idea that we need to take our embodied existence as particular human beings seriously; and that doing so requires we explore and pay attention to how people experience their embodiment. Thus the purpose of this paper is to consider the thesis that we need to pay attention to people’s embodiment. In other words, the goal is to consider both the arguments for *why* we ought to take people’s embodiment seriously, and *what* taking people’s embodiment seriously should look like in ethical theory and political philosophy.

To this end, in section 2, I consider the reasons why we ought to take embodiment seriously in ethical theory and political philosophy. I distinguish between ‘weak embodiment claims’ (i.e. those which are compatible with a wide variety of ethical theories), and ‘strong embodiment claims’ (i.e. those the acceptance of which requires rejecting some standard presuppositions of ethics and political philosophy). The weak embodiment claims I consider are arguments for taking embodiment seriously based on the values of autonomy, harm-avoidance, and fairness. The strong embodiment claims I consider are: (i) the claim that taking embodiment seriously requires taking a relational approach to ethics (e.g. an ethics of care or a vulnerability approach); and (ii) the claim that taking embodiment seriously requires we adopt a particularist methodology in ethics and eschew the use of ethical principles.

In section 3 I argue we ought to reject the second strong embodiment claim, i.e. the idea that taking embodiment seriously requires rejecting universal ethical prescriptions. Insofar as the normative prescriptions of the ethics of care or vulnerability approaches are dependent on accepting the truth of particularism, rejecting particularism will require rejecting those claims too. This, however, does not mean that all the insights of the ethics of care or vulnerability approaches need to be rejected. As we will see in section 4, in recent years the insights of the ethics of care have been reformulated in terms of principles, showing that accepting the idea that we are all vulnerable and in need of care doesn’t necessarily commit one to adopting a particularist metaethics. The ethics of care and vulnerability approaches, therefore, are not as fundamentally opposed to either liberal political philosophy in particular or universalist approaches to ethical theory in general (as is sometimes made out). Instead, there is ample room for reconciliation. This, I suggest, is good news, as it opens up the possibility that the insights of the ethics of care and vulnerability approaches can be brought together with the insights of liberal political philosophy and more mainstream approaches to ethical theorising.

## Why Care About Embodiment?

Why should we take embodiment seriously in ethical theory and political philosophy? Why should we care how people experience their embodiment when it comes to making normative judgements? In this section Iexamine a number of potential reasons for taking embodiment seriously. I argue the various claims can usefully be divided into strong and weak embodiment claims. The difference between them is this: whereas weak embodiment claims are compatible with a wide variety of ethical theories, accepting strong embodiment claims, as will become apparent later, requires a more wholesale overhaul of how ethics is practiced. Let us start with the weak embodiment claims.

### Weak Embodiment Claims

The first reason we should care about people’s embodiment is that how we are embodied can have effects on our ability to exercise autonomy in the pursuit of our goals. Our bodies are the medium through which we act on the world, and what our bodies are like has pervasive, wide-ranging, and long-lasting effects on what we are able to do.^34^ Our autonomy can be impaired by illnesses, either acute or chronic. Having a headache, gastrointestinal problems, a fever, or a respiratory infection can make it harder to pursue one’s plans (at least temporarily). In the words of S Kay Toombs, ‘[r]ather than functioning effectively at the bidding of the self, the body-in-pain or the body-malfunctioning thwarts plans, impedes choices, renders actions impossible.’^35^

In the case of chronic illness or disability, the impairments to our autonomy occur over a longer period. Chronic illness can have the effect that quotidian activities which could be performed easily prior to illness become ‘an explicit task, requiring thought, attention, and a pronounced effort.’^36^ Havi Carel, for example, describes her experience of breathlessness thus:

A distance I would once call ‘near’ or ‘a day’s walk in the countryside’ is now ‘far’ or ‘impossible’. Small tasks like carrying groceries home or lifting a child require preparation, pauses, rest, and cause fatigue. Everything is hard. Everything is far. Everything is strenuous.^37^

Individuals who use assistive devices such as wheelchairs, for example, may find they are dependent on others for assistance (e.g. getting shopping down off high shop shelves)^38^ or that their plans are frustrated by inaccessible urban design.^39^ Use of a visible prosthesis or assistive device can also lead to others perceiving the user as dependent and treating them in accordance with this perception, which can be further disempowering.^40^ S Kay Toombs, for example, reports that:

On those occasions when I use a wheelchair, strangers invariably address themselves to my husband and refer to me in the third person. ‘Would *she* like to sit at this table?’ ‘What would *she* like to drink?’ and so forth.^41^

Finally, as Iris Marion Young argues in ‘Throwing Like A Girl’, how we are taught to use our bodies can be disempowering and thwart our capacity to effectively assert our will in the world.^42^ Young argues that women socialised in patriarchal societies are taught to use their bodies in ways which fail to ‘make use of the body’s spatial and lateral potentialities’^43^ which causes women to ‘greatly underestimate our bodily capacity’^44^ and leads to an inhibited intentionality.^45^

Inquiring into how people experience their embodiment is, thus, important because it can reveal ways in which autonomy is impaired or frustrated which might otherwise be overlooked. For instance, until one enquires into the lives of people with type 1 diabetes (T1D), it is difficult for a person without diabetes to understand how much work is required to maintain safe blood-glucose levels. Standard treatment for people with T1D consists of patients self-injecting insulin multiple times a day.^46^ People need to adjust the dosage of insulin they are injecting in response to blood-glucose levels, which are measured by pricking one’s finger. Based on this information, people then need to adjust how much insulin they should inject based on up to 42 other factors;^47^ including the type of meal eaten, level of exercise, or whether they are ill. Whilst digital diabetes care technologies such as continuous glucose monitors and insulin pumps represent an improvement over traditional treatment modalities, they nevertheless fail to completely alleviate the mental and physical burden of managing T1D for those with the condition.^48^

Something similar occurs with prostheses. Until one becomes acquainted with first-person accounts of what using a leg prosthesis is like, it is easy for non-users to assume that a prosthesis will unproblematically enhance people’s autonomy by enhancing their mobility. However, the reality of prosthesis use is much more complex. Learning to use a prosthesis requires a process of adjustment to the device, as well as the relearning of once familiar everyday activities.^49^ Users have to adopt a controlled diet to avoid changes in the shape of the residual limb,^50^ adjust the fit of the prosthesis throughout the day,^51^ and adopt a new gait to balance their centre of gravity.^52^ The need for this continual process of adaptation can be experienced as frustrating as it interferes with daily life. Having to pay conscious attention to their prosthesis makes simultaneously engaging in other activities very difficult (e.g. walking and talking with a friend).^53^

The point of all of this is that understanding the burdens and benefits particular forms of embodiment (and experiences thereof) can have on people’s capacity to exercise autonomy is a necessary precondition to successfully mitigating these effects. Consequently, if we care about people’s effective ability to exercise their autonomy and live their lives in a manner of their own choosing, then we ought to care about how people are embodied.

A similar argument can be made about harm. As many harms are (at least partly) experiential, inquiring into how people experience their embodiment can help us understand the character and magnitude of harms people are experiencing. Consider, for example, painful experiences as a form of harm. The fact an experience is painful is often part of the explanation for why that experience is harmful. Now, we all know the pain of others cannot, literally, be felt. Although we might be able to perceive that someone is in pain through observing their movements or facial expressions, acquiring knowledge of what the pain of another person is like (e.g. its intensity or character) generally requires asking them to describe it.^54^ If some harmful states of affairs are harmful in virtue of their painfulness, understanding the nature and magnitude of the harm will require inquiring into people’s first-person experience of their embodiment.

Perhaps the best illustration of the importance of taking people’s experiences seriously in assessing the harmfulness of a state of affairs is the existence of the disability paradox. The disability paradox is the term given to the mismatch between disabled people’s self-assessment of their quality of life (which can be high) and the assessment made by non-disabled people (generally lower).^55^ The disability paradox powerfully illustrates how difficult it can be to estimate how other people experience their embodiment from afar.^56^ If we want to accurately understand the harms people are experiencing, we need to inquire into people’s first-person perspective.

Having an accurate understanding of how harm is experienced is important for two reasons. First, if the harm in question was inflicted wrongfully, understanding the nature of the harm is important to determining the magnitude of the wrong inflicted. Second, understanding the nature of the harm is important in understanding how to mitigate its effects. In the words of Fredrik Svenaeus:

it means very little to say that a patient should be helped and not harmed if you have not developed an understanding of the more precise ways in which this particular person is suffering.^57^

A third way in which embodiment matters in ethical theory and political philosophy is when it comes to determining the extent and nature of both people’s entitlements under a cooperative scheme and their duties towards the scheme. According to fair-play theories of justice, those who benefit from a mutually advantageous cooperative venture have an obligation to contribute their fair share of the costs of running the scheme.^58^ What constitutes a fair share of duties for a person might plausibly be influenced by how they are embodied. Some ways of being embodied make particular activities especially burdensome, either permanently or temporarily. In a subset of these instances, the level of burdensomeness leads us to waive the duty to contribute to the cooperative scheme. In others it might lead us to reduce the share of duties for those who are less able to contribute and redistribute those duties to those with greater ability to satisfy them.^59^ This sort of reasoning can explain why we ought to make reasonable accommodations for some individuals with disabilities at work and, where a person’s condition makes work impossible or overly burdensome, provide for their support through a social benefit system.

Relatedly, an acknowledgement of the burdens imposed by particular forms of embodiment is important to determining what entitlements people have under a fair cooperative scheme. Due to how some people are embodied, they need a greater share of resources to achieve a similar level of wellbeing or have access to similar opportunities than people who are embodied differently.^60^ It is widely accepted that certain forms of disability make particular activities more costly, a phenomenon known as the ‘disability price tag’.^61^ If someone, for instance, has limited mobility, travel becomes more expensive. Whereas someone with full use of their legs could walk to an appointment, people with limited mobility may require a taxi. Whereas someone who can bend down easily and climb ladders can clean and maintain all areas of their house, people who are unable to do this will require assistance (which will often have to be paid for).

Understanding how people are embodied is, thus, crucial to understanding what a fair allocation of the burdens and benefits of social cooperation is. If we care about ensuring fairness, we need to inquire into people’s embodied experiences. The three reasons for taking embodiment seriously outlined above seem to be compatible with most ethical theories. For instance, the concerns about the effects of people’s embodiment on the exercise of autonomy ought to matter to any theory that places value on autonomy (either directly or indirectly). As such, these concerns should, for instance, be capable of being accommodated by both deontologists who conceive of autonomy as intrinsically valuable and consequentialists who believe that autonomy is instrumentally valuable in virtue of its contribution to other goods such as wellbeing or happiness.

Accepting these weak embodiment claims also doesn’t require we give up on the idea of the self as a rational agent as the holder of rights and bearer of duties, even if it does have implications for how we cash out some of these rights and duties. Since these views don’t seem to presuppose any particular controversial ethical theory and can be interpreted as particular ways of cashing out various theories, weak embodiment claims could plausibly be subject to an overlapping consensus.

### Strong Embodiment Claims

Alongside these weak claims we also find a series of stronger claims which, as we will see, are not compatible with a wide variety of ethical theories. Accepting these arguments, therefore, will require a more wholesale rejection of traditional ethical theorising. Unlike the weak embodiment claims outlined above, the insights they contain cannot simply be incorporated into the specification of both deontological and consequentialist ethical theories.

The first ‘strong embodiment claim’ that is sometimes found in the literature is the idea that taking embodiment seriously forces us to take a relational approach to ethics which gives centre stage to our inevitable dependence on each other.^62^ Whereas traditional ethical theorising conceives of individuals as independent agents, focusing on embodiment reveals that ‘we are located in relations that transform the natural and social worlds in which we live. It is within networks of interdependence that we can affect the actions of other people.’^63^ How we experience our embodiment is thus shaped by cultural discourses and relations of power.^64^ Taking embodiment seriously, therefore, requires we pay attention to the relationships between individuals and the social structures that shape these relations.

Dependence and vulnerability theorists claim, contrary to what the liberal view of the self is said to presume, that our lives are marked by substantial dependence on others.^65^ We are born vulnerable and dependent, and we often die that way too. Between these two poles, our lives can be marked by periods of illness and injury, which also render us dependent and vulnerable.^66^ We are autonomous, at most, for a brief period limited by the dependencies of childhood and old age. Even during that period we are sustained by relationships of dependency with others without which our autonomy would be limited. When we do achieve autonomy, then, it is because others have sustained us in periods of vulnerability.^67^

This approach finds expression in the work of theorists such as Alasdair MacIntyre^68^ and Martha Fineman,^69^ who argue that being an embodied being ‘carries with it the ever-present possibility of harm, injury and misfortune, from mildly adverse to catastrophically devastating events’.^70^ Importantly, vulnerability is not limited to certain groups of people, it is ‘a universal, inevitable, enduring aspect of the human condition’.^71^

Taking our embodiment seriously on this account requires focusing political institutions around ensuring resilience to vulnerability. On a vulnerability account, care and dependency are not private affairs best left to families and markets.^72^ The responsibility to take care of dependents must be shared amongst all members of the political community.^73^ In other words, a vulnerability approach will require public support for those who care for others. In practical terms, this will require providing cash benefits and benefits in kind (e.g. public housing and food stamps) to carers;^74^ ensuring access to affordable childcare for parents;^75^ regulating the labour market to ensure better job security, better wages, and safe working environments;^76^ providing paid sick or carers leave;^77^ workplace accommodations such as flexitime and job shares for carers and parents;^78^ ensuring universal access to healthcare, housing, education, and a minimum income;^79^ and ensuring substantive (as opposed to merely formal) equality of opportunity for all.^80^

Another example of an approach, which takes our relationality and dependence seriously, is the ethics of care. As the name suggests, the ethics of care takes care to be a central notion. Caring involves providing for people’s needs, helping them sustain and develop their capacities, and relieving suffering.^81^ Caring requires attentiveness to people’s needs, responsiveness in the satisfaction of these needs, and respect for dependent people’s equal moral value.^82^ The ethics of care differs from universalist accounts of ethics (i.e. those that hold that ethical prescriptions apply universally) in a number of respects. First, it focuses on the importance of relationships and conceives of the self as fundamentally relational.^83^ Liberal theories based on the independent autonomous self, care ethicists argue, ‘miss the moral importance of actual, caring relations.’^84^ The reason these relationships are important, care ethicists argue, is because we are all dependent at some points in our lives. We have all received care as children, and we will likely all require care at some point in our adult life as we age. Without this care at various points in our lives, we would not ‘grow up’ to be the independent, autonomous self that liberalism focuses on.^85^

Second, an ethics of care holds that moral deliberation is not exclusively about rationality, it also requires empathy and emotional responsiveness to others.^86^ The emotions help us ‘understand what we ought to do’ and serve to motivate moral action.^87^ Third, unlike universalist liberal ethics and political philosophy, an ethics of care does not focus primarily on rights.^88^ Instead, it takes other people’s needs within relationships as the starting point for determining what one ought to do.^89^ For care ethicists, our obligations to care are founded upon and justified by our common dependency.^90^

According to a care ethics account, care is not purely a personal value.^91^ Many care ethicists argue that taking the fact we are dependent and in need of care seriously has political consequences.^92^ Precisely what these political consequences are, however, is not always spelled out in much detail.^93^ Eva Feder Kittay, for instance, says that an ethics of care requires that wider society support relations of dependency work without saying much about what form this support should take.^94^

The care ethicist who has been most explicit is Daniel Engster. In *The Heart of Justice* and *Justice, Care and the Welfare State*, Engster argues that a government conforming to the prescriptions of care ethics would provide military and police protection, a clean and safe environment, sanitary water and sewage processing, basic infrastructure goods such as roads and bridges, basic rights to physical integrity and security, protections against cruel and unusual punishments, protections from arbitrary arrest, rights to fair trials,rights against discrimination, prenatal care for women, income subsidies for poor families with children, universal education, access to healthcare, sick-leave, pensions and care facilities for the elderly, subsidies and workplace accommodations for disabled people, personal assistance for those who need them, unemployment benefits, housing vouchers for those on low incomes, flexible workplace scheduling, occupational safety standards, and legislation covering maximum work hours.^95^

Another, related, strong embodiment claim that is sometimes found in the literature is that focusing on embodiment should lead us to abandon the pursuit of universalisable ethical prescriptions.^96^ Although not all embodiment theorists hold this view, and it has fallen somewhat out of favour in recent years, the view is worth considering for two reasons. First, it is common enough in the literature that to ignore it would be to overlook a significant aspect of what some embodiment theorists claim taking embodiment seriously requires. Second, if true, it would require a radical overhaul of how ethics is practiced.

Among those who hold that taking embodiment seriously requires adopting a particularist approach to ethics are Drew Leder, Leon Kass, Rosalyn Diprose, Moira Gatens, Virginia Held, Nel Noddings, Rachel Tillman, and Jeremy Wisnewski. 97 The forms of particularism these authors advocate varies. Sometimes the term particularism is not particularly well explained, leaving it to the reader to determine what it means.^98^ More trenchant particularists hold that there are no moral principles at all, that principles are not useful moral guides, and that morality does not consist in applying principles to cases. Weaker forms of particularism hold that although principles may be discoverable, morality can be understood without principles, which are at best ‘crutches’ to help people comply with morality.^99^

However, to the extent that we can ascribe a common understanding amongst theorists, we can say that particularists hold that, instead of thinking about ethics in terms of the correct moral principles, we ought to focus on the particulars of the situation, focusing on the actions of individuals in their particular circumstances, taking into account the moral sensibilities, affections, embodied first-person perspectives, habits, and customs of particular moral agents.^100^ On this view, ‘we ought not to rely on moral principles in moral thought and judgements because they provide poor guidance for doing the right thing’.^101^ Instead of illuminating the situation, a focus on rules ‘can obscure an entire realm of moral reality and moral obligation – it can lead us to think of rules when we should be thinking of people.’^102^ Although it could be argued that this understanding of particularism sits on the more trenchant side of the spectrum, I believe it is, nonetheless, justified. This is because the most developed accounts of particularism tend to go beyond the weak claim that morality could, *in theory*, be understood without principles, and instead make the stronger claim that principles lead us astray and obscure important considerations.^103^

A number of different reasons are given to justify the claim that universalizable prescriptions are poor moral guides. The first is that principles are not sensitive enough to context.^104^ The search for generality leads to glossing over meaningful differences, including differences about how different people are embodied; potentially biasing one’s answer to the case.^105^ The second reason is that universalist ethics that search for principles assume that reasons always function in the same way in different contexts. Contra this, the particularist claims that a reason can make a moral difference in one case, but not in another.^106^ Reasons, on this view, have an irreducibly context-dependent valence.^107^ R can be a reason in favour of doing X in one case, and a reason against doing X in another.^108^ To illustrate: the fact I have borrowed a book from someone is generally a reason to return it to them. However, if the book was stolen from the library, it is a reason not to return it to them and to return it to the library instead. Moreover, according to the particularist, this variability of reasons ‘cannot be cashed out in finite or helpful terms.’^109^ On the basis of these sorts of cases, particularists hold that:

one cannot extract from one case anything that is guaranteed to make a difference to another. They recommend keeping one’s eyes firmly fixed on the case before one rather than trying to squeeze an answer to one problem out of the answer to another.^110^

Rejecting universalisable ethical prescriptions leads to the rejection of a wide variety of ethical and political theories including deontological approaches such as Kant’s, consequentialist ethics, and contractarian ethics.^111^ In this sense, they are different to the weak embodiment claims outlined above which can be incorporated into both consequentialist and deontological approaches to ethics. If, however, taking embodiment seriously requires rejecting universalist ethics, this move is unavailable, as both consequentialist and deontological views are committed to universal ethical prescriptions. As such, if we accept the strong embodiment thesis outlined, taking embodiment seriously will require a wholesale overhaul of ethical theory.

## Against Particularism

In this section I  will argue that we ought to reject the claim that taking embodiment seriously requires we reject universalist ethical prescriptions. Insofar as the prescriptions of both the ethics of care and vulnerability approaches are necessarily based on a particularist meta-ethics, this will also involve rejecting some (but not all) aspects of these approaches. Where the claims can be reformulated without relying on a particularist account of moral reasoning, the central insights of both approaches can be salvaged. In section 4 I outline how a number of the insights of both vulnerability approaches and the ethics of care can be reformulated in such a way as to avoid relying on a particularist metaethics.

With this caveat in mind, it is time to turn to the argument against particularism. I take a two-pronged approach to arguing against a particularist approach to ethics. First, I outline a series of general problems with particularist accounts of ethics which ought to temper anyone’s enthusiasm for rejecting universalist accounts of ethics. Having done this, I will then challenge the inference that taking account of people’s embodiment requires adopting a particularist account of moral reasons by showing that universalist ethics can, in fact, take the facts of our embodiment into account, including the fact of universal vulnerability and the ever-present possibility of dependence on others.

Let us turn to the first line of argument: objecting to particularistic accounts of ethics. The first problem with particularistic accounts of ethics is that it is difficult to understand how we can justify our decisions to each other if reasons are variable in the way particularists claim they are. If moral reasons flip valence in an un-codifiable way, standard forms of argument in ethics are ruled out. Much ethical argument proceeds on the basis of analogies between cases.^112^ The problem is that these arguments only carry force if we can assume that a given reason will function in much the same way in another comparable case. Therefore, particularists are forced to deny the validity of many arguments from analogy.^113^

Second, the idea that reasons can function in opposite ways in different cases seems to challenge the basic idea that rational thought requires consistency. Third, and relatedly, moral particularism makes it hard to avoid special pleading.^114^ Reasons with invariable (or fixed) valence across cases can play a motivational role, keeping us on track ‘when we are likely to go astray through partiality to self or friends’.^115^ If reasons have the kind of variable valence Dancy thinks they have, however, it is difficult to see how they can play this role. This is a genuine worry because:

one can always find some difference between this act and a plain duty, and there seems to be no way, within the resources available to particularism to prevent such differences being appealed to by those who, in bad faith, want to let themselves off the moral hook.^116^

If we could assume that reasons always functioned in the same way in different cases, we could challenge special pleading on the grounds that the feature appealed to doesn’t make a difference in other cases. However, this response is not available to particularists.^117^

Having given some preliminary reasons to be sceptical of particularist accounts of moral reasoning, it is time to move on to the second line of argument: challenging the inference that taking embodiment seriously requires adopting a particularist metaethics by showing how general ethical prescriptions can take account of context dependent factors in general, and facts about embodiment in particular.

Particularists are undoubtedly right that some formulations of particular principles might be poor moral guides in virtue of the fact that they ignore context dependent features such as the facts of people’s embodiment. Generalists can agree with this basic point, as they are not committed to defending the validity of poorly constructed principles. Showing that a poorly constructed principle yields wrong answers does not demonstrate that all will and, hence, does nothing to undermine generalism. Neither are generalists committed to ignoring context dependent factors such as facts about how people are embodied or not ‘focusing on the case’ (as is sometimes claimed).^118^ Reasoning in terms of principles always involves paying attention to the case to see whether our principles apply, what they require in that particular situation, and how they could be implemented.^119^

The method of reflective equilibrium, which is both perfectly compatible with generalism about reasons and widely used by philosophers, aims to do just this.^120^ ‘Focusing on the case’ is achieved by identifying our considered judgments about cases, which are then either modified in light of more general principles or used as the basis for modifying the general principles.^121^ The context-dependent facts (such as facts about how particular individuals are embodied) are taken into account during this process of refining principles to ensure consistency and coherence amongst them and our considered judgements.^122^

There is thus nothing barring generalists from incorporating context, including differing embodiment, into the application of principles to cases. As argued above, the facts of embodiment are important when cashing out the magnitude of harm, identifying threats to (and ways of supporting) autonomy, and determining the extent of people’s duties. Particularists are wrong that principles necessarily ignore context. Given that principled approaches can take account of contextual factors (including facts about how people are embodied) into the application of principles, the inference that taking embodiment seriously requires adopting a particularist meta-ethics is erroneous. Taking embodiment seriously is open to both generalists and particularists. The debate isn’t, therefore, whether to take account of context, but how, and to what extent to do so.

Generalists argue that we need to codify how context influences the application of principles and incorporate this into the principle itself, making the principle more complex. Particularists will retort that, in light of the variability of reasons, we can’t take account of context in any general way. Situations where reasons do not function as they might do in other cases cannot be codified in advance. The most we can do is have a (potentially very long) list of features that might make a difference.

The most persuasive response to this problem is Pekka Vayrynen’s argument that we can synthesise the list of exceptions by incorporating the ‘normative basis’ of the principle into the construction of the principle itself. What the normative basis is will depend on one’s substantive ethical theory and, hence, is subject to disagreement. What all normative bases have in common, however, is that they establish some sort of relation (promoting, honouring, respecting, protecting) between a non-moral feature (F) of an action x (e.g. x is an act of returning a book) and a moral evaluation (M) (e.g. x is pro tanto right). The appropriate relation between F and M will be something of moral significance (e.g. returning property to its rightful owner) which gives us reason to do F. Incorporating this relationship into the principle itself allows us to propose ‘hedged principles’ of the form:

For any x, if x is F, then x is M in virtue of being F, provided that x instantiates the designated relation for F and M.^123^

Or, in plainer language: If I am returning a book, then what I am doing is pro-tanto right provided that the act of returning the book in this case is a way of returning property to its rightful owner. Understood in this way, principles can be appropriately general, whilst being hedged and sensitive to context.

To sum up, we ought to reject the idea that taking embodiment seriously requires giving up on ethical principles in favour of a particularist approach to ethical theorising. When appropriately hedged, principles can take adequate account of context. Information about people’s embodiment, for example, will be relevant to understanding the harms people experience, how their autonomy might be supported, and the extent of their obligations towards others and the obligations of others towards them.

At this point it could be objected that taking account of people’s differing embodiments isn’t only about taking account of contextual factors that affect how we cash out principles in moral reasoning.^124^ Although how people are embodied is an important part of the context within which we apply general principles, how people are embodied could also be seen as influencing people’s subjectivities. This includes factors such as how they perceive situations; interpret their relevance, and (ultimately) what the correct interpretation of concepts such as autonomy, harm, and fairness ought to be. If this is the case, the objection continues, showing that facts about how people are embodied can be taken into account as part of the context within which we apply principles is not enough. Taking embodiment seriously will also require taking into account how people’s divergent experiences affect the interpretations of concepts such as autonomy, harm, and fairness and the value placed on them.

Although the focus of the preceding discussion has been on how the facts of embodiment affect the application of principles, the arguments in this section are not intended to rule out the fact that people’s embodied experiences may both lend support to particular conceptions of autonomy, harm, or fairness, and raise questions about how the relative value of supporting autonomy, preventing harm, and ensuring fairness. Disputes about the correct interpretation of normative concepts, their relative value, and how to take into account divergent perceptions and interpretations of events are, after all, the bread and butter of ethical theorising. As we will see in section 4, taking embodiment seriously requires placing less emphasis on autonomy *per se*; instead, placing greater emphasis on the facts of embodiment (including our inevitable (inter-)dependencies) than is commonly the case in liberal approaches. The important point for now – as far as this section goes – is that engaging in these disputes does not require adopting a particularist meta-ethics.

## Taking Account of Embodiment without Particularism

In Section 2.2 we saw that care ethicists such as Daniel Engster argue that a care ethics approach can be used to justify many of the institutions of the welfare state, including police protection, courts, public education and healthcare (amongst others). Similarly, Martha Fineman argues that taking a vulnerability approach reveals the importance of state support for caretakers and parents, workplace accommodations aimed at enabling people to care for those who depend on them, and universal access to important basic services such as healthcare and goods such as housing.

As we also saw above, sometimes the demands of the ethics of care are justified using a particularist metaethical framework. If the arguments of the previous section are correct, conclusions justified by appeal to particularism are on shaky ground. The task of this section is to show that, contra what some care ethicists and vulnerability theorists claim, the central insights of these two views can be reinterpreted in universalist terms, allowing them to evade the objections to particularism developed in section 3. Although accepting these claims might require modifying or limiting some aspects of standard accounts of liberal justice, vulnerability and care ethics approaches are not as opposed to liberal accounts of justice as is sometimes made out.^125^ Instead, as Daniel Engster and Stephanie Collins’ recent work on the ethics of care shows, if the insights of the ethics of care can be divorced from particularist metaethical baggage, there is ample room for reconciliation.^126^

This, I suggest, is good news for anyone committed to the importance of taking account of how people are embodied in ethical theory and political philosophy for two reasons. The first reason is that, if the insights of care ethics and vulnerability approaches can be made compatible with liberal accounts of justice, theorists committed to taking embodiment seriously can free themselves of the need to propose alternative accounts of, for example, why central liberal rights such as freedom of speech and due process are important, or how these rights can be justified by the value of care or in virtue of them enhancing resilience to vulnerability. Even if, with the appropriate mental gymnastics, these could be justified in terms of care or vulnerability,^127^ by adopting an approach on which care and justice are seen as complementary, these justificatory tasks can be fulfilled by drawing on other values (e.g. autonomy or equality), something which might offer more straightforward justifications for these rights.

The second reason is that, if the insights of the ethics of care can be reformulated in non-particularist terms, accepting these claims carries less metaethical baggage. Consequently, there are fewer reasons to reject the central insight that we ought to take people’s experience of embodiment seriously. Instead of being tied to partisan disputes about the nature of moral reasons, the central insights that we are all dependent, vulnerable, and in need of care might plausibly be seen as the sort of considered judgement we want principles to explain or accommodate in the process of reflective equilibrium.^128^ In other words, they might be insights that could be subject to an overlapping consensus open to theorists who disagree on the ultimate foundational questions of moral theory. With this preamble in mind, let us turn to the main task of this section: showing that the insights of an ethics of care and vulnerability approaches can be captured without relying on a particularist methodology.

As we have seen, some early care ethicists such as Nel Noddings and Joan Tronto explicitly reject the use of principles in moral reasoning.^129^ This is also true of some vulnerability theorists such as Catherine Mills and Judith Butler.^130^ However, this is not the case with all care ethicists. Daniel Engster and Stephanie Collins, for example, both formulate their versions of care ethics in terms of principles without them being vulnerable to the accusations that they fail to focus on the case, that the principles they use are poor moral guides, or that they gloss over relevant differences.

In his interpretation of care ethics Daniel Engster makes liberal use of ethical principles to justify duties of care.^131^ In *Justice, Care, and the Welfare State*, for instance, Engster writes: ‘When we refuse to respond to others’ needs for care, we violate a basic moral principle that we ourselves have implicitly endorsed in claiming care for ourselves: that capable individuals should care for others in need when they can.’^132^ In other words, not responding to others’ needs through care is a form of performative contradiction because ‘our own claims for care thus logically commit us to extend care to all other beings who necessarily depend upon human care for their survival.’^133^

Crucially, the argument that we violate a moral principle by not providing care in situation X when we have claimed it in situation Y depends on reasons not being variable in the way that particularists hold they are. If reasons have variable valence, the fact we claimed care in situation X has no direct implications for what we ought to do in a similar situation Y as ‘one cannot extract from one case anything that is guaranteed to make a difference to another.’^134^ Reformulating the ethics of care in terms of principles therefore has the advantage that it increases the normative force of the argument for the importance of care by reducing the potential for special pleading aimed at sidestepping the demand that we provide care to those who need it.

As well as making the case for care stronger, Engster’s framing of the ethics of care in terms of general principles does not gloss over relevant differences or fail to focus on the case. On the contrary, the policy proposals Engster derives from the principle that we owe each other care are both empirically informed and tailored to the realities of contemporary social life. For instance, when arguing that care requires universal healthcare provision, Engster uses empirical data to argue that publicly regulated multi-payer systems are superior to both single payer and market systems because they ensure easy access to care, equity in care provision, good acute and chronic care outcomes, funding for preventative care, and high levels of patient satisfaction and trust.^135^ Far from glossing over the complexities of the case, or failing to notice difference, Engster’s cashing out of the principle of care is highly context specific.

In her book *The Core of Care*, Collins also proposes a principled form of care ethics. Collins argues that the insights of care ethics can be specified, unified, and justified by a principle of dependency,^136^ which holds (roughly) that ‘agent A has a moderately strong dependency duty to take measure M when A’s most efficacious measure for fulfilling an important interest is sufficiently likely to fulfil the interest and would realise positive expected value regarding agent and dependent.’^137^ As with Engster’s interpretation of the ethics of care, Collins’ use of the principle of dependency is perfectly compatible with taking account of context-dependent features of the case at hand. When fleshing out the rough characterisation of the principle of dependency cited above, Collins includes a variety of context-dependent factors which influence her analysis of whether the duty applies. For instance, when discussing whether someone’s fear of water means that they do not have a duty to save a drowning child, Collins distinguishes between cases in which the person’s fear is incurable (in which case they don’t have a duty) and cases where the person could have taken reasonable steps to cure their fear (in which case they are morally deficient for failing to do so).^138^

As these examples show, care ethicists should not be opposed to using principles as justifications for the duties of care they argue for even if they are sceptical of their role in ensuring caring actions in particular circumstances. Nevertheless, the fact that some care ethicists recast their arguments in terms of principles, however, does not yet show that these principles are compatible with standard liberal approaches to justice. It might still be the case that the principles that care ethics or vulnerability approaches put forward are so distinct from the principles put forward by standard accounts of liberal justice that no reconciliation between the different views is possible.

Recent work in care ethics, however, belies this interpretation. Although some care ethicists such Nel Noddings (in her early work), Carol Gilligan, and Michael Slote argue that an ethics of care can be seen as a theory of the whole of morality and political philosophy,^139^ this view has fallen out of favour.^140^ Care ethicists such as Stephanie Collins, Daniel Engster, and Virginia Held see care ethics as a complement to liberal accounts of justice.^141^ Care and justice, on this view, are not fundamentally opposed.^142^ Although it is true that we are all often dependent and in need of care at various points in our lives, we are also sometimes autonomous and need our independence guaranteed and supported.^143^ Ignoring either of these basic facts about our lives will give us an incomplete picture. If we want to take embodiment seriously, we need to take account of both of these features of our embodied experience.

There are various ways in which this could be done. On a relational account of autonomy, dependence and independence are not in an antagonistic relationship. Relations of dependence between people don’t only enable care, they also support and enable independence. On relational accounts of autonomy, therefore, there is no fundamental tension; both aspects of our lives must be taken into account in the name of autonomy itself. On non-relational accounts of autonomy, on the other hand, dependence and independence are genuinely contrasting states. Taking both into account on a non-relational conception, requires limiting autonomy in the name of the value of caring for dependents (and vice versa). Which of these two solutions is to be preferred is beyond the scope of this paper. The important point here is that we shouldn’t ignore either aspect of our lives.

So far in this section I have argued that the central insights of care ethics and vulnerability approaches can be expressed in terms of principles and made a case for seeing care ethics and vulnerability approaches as complements to liberal accounts of justice. What is still to be addressed is the question of what standard approaches can’t get us. As we have already seen, care ethicists and vulnerability theorists hold that the facts of dependency have generally been overlooked in political philosophy and ethical theory. The question is, how should standard approaches to liberalism be modified to take better account of these facts?

As we saw above, care ethicists and vulnerability theorists argue that a number of political consequences follow from taking our embodiment seriously. These include the need for well-funded welfare services, healthcare, workplace accommodations for disabled people (among others). The problem for care ethicists and vulnerability theorists is that many of these policies can also be justified in light of other ethical theories and political philosophy. To give just two examples, basic rights such as freedom from arbitrary arrest can be justified by appealing to the value of freedom. Prohibitions on discrimination and universal provision of education and healthcare can be justified by appeal to the importance of equality of opportunity.

If this is the case, what does taking a care ethics or vulnerability approach add? If many of their prescriptions can be justified in light of other approaches, in what sense can it be said that these approaches neglect people’s embodiment? The best answer to this question is that, although many of the facts of embodiment can be taken account of by other theories, what they cannot do is place the amount of emphasis on them that an ethics of care and/or a vulnerability approach does.

Daniel Engster, for example, argues that arguments for universal healthcare that rely on the idea that health is important for equality of opportunity will fail to justify universal provision of healthcare because, as social determinants of health research has shown, healthcare has a relatively limited impact on people’s health. If equality of opportunity is the goal, the money is likely best spend elsewhere. Once we realise that part of the importance of healthcare is the care it provides people, we are better placed to justify universal provision.^144^

Similarly, even if care for the elderly, schooling, and welfare programmes can be justified by appeal to other values such as autonomy or equality of opportunity, they might not justify the amounts or levels of these services that a care ethics approach might.^145^ Similarly, justifications for welfare programs that draw on justice might also have little to say about how these services are delivered. In Virginia Held’s words, ‘even if the requirements of justice and equality would be met by a certain program, of payments, let’s say, we could still find the program callous and uncaring if it did not concern itself with the actual well-being (or lack of it) brought about by the program.’^146^ Justice approaches might have little to say, for instance, about payments being provided grudgingly or recipients being stigmatised in the process; concerns which could feature more prominently in an ethics of care approach.

In other words, standard liberal approaches will offer a weaker, less fullhearted and more-roundabout justification than an ethics of care.^147^ Compared to liberal justice accounts, an ethics of care is playing on home turf when the focus is on welfare, healthcare, and care services. This is where seeing liberal justice and care ethics as complementary approaches, neither of which covers the whole of morality, comes into its own. This makes it possible for us to give the best, most coherent, justification for each of the institutions and policies of the welfare state, with some policies being justified by the values of autonomy, harm, and fairness; and others justified by the value of care.^148^

## Conclusion

As we have seen, it is sometimes suggested that liberal political philosophy and mainstream ethical theories pay insufficient attention to the fact that people are embodied. Liberalism’s abstract conception of the self is said to prioritise independence and autonomy, obscuring our inevitable and universal vulnerability as embodied beings. Similar charges are often levelled against mainstream deontological and consequentialist ethical theories. As these views are committed to universalisation and abstraction in ethical argument, they are purportedly forced to ignore the importance of contextual knowledge in determining what is right and/or good.

The task of this paper has been to consider what taking embodiment seriously in ethics and political philosophy requires. I have argued that, contrary to what is sometimes claimed, both liberalism and mainstream ethical theories can, in fact, take account of people’s embodiment. I have shown that, so long as a political or ethical theory makes use of the concepts of harm, autonomy, or fairness, the theories (and by extension those who subscribe to them) are committed to taking people’s embodiment seriously. In particular, without taking account of how people are embodied it is impossible to fully ascertain the sorts of harms people experience, what their entitlements and duties under a cooperative scheme are, and what supporting or protecting people’s autonomy requires. Far from presupposing a disembodied view of the self, ethical and political theories that make use of the notions of harm, autonomy, and fairness cannot be cashed out without taking account of people’s embodied nature.

I have also argued that, contra what particularists hold, taking people’s embodiment seriously does not require eschewing the use of principles in ethics and political philosophy. If giving our embodiment due regard did require adopting particularist approaches to ethics, critics would indeed be right that liberal political philosophy and mainstream ethical theories would be incompatible with taking sufficient account of people’s embodiment. This, however, is not the case. Although it is true poorly formulated principles may be bad ethical guides and obscure important contextual features, I have argued that if principles are appropriately hedged, principles can be both suitably general and sensitive to contextual factors. As a consequence, taking embodiment seriously needn’t require a wholesale rejection of liberal approaches to justice in favour of an ethics of care or vulnerability approach.

Instead of conceiving of care ethics and vulnerability approaches as complete alternatives to liberal approaches to ethics, I have argued we ought to see them as complements to each other. Although it is true that we are all, over the course of a lifespan, vulnerable and dependent on care from others, we are also sometimes autonomous. Both these features of our lives need to be taken seriously. Doing so will require both liberal approaches to justice which focus on the importance of freedom and autonomy, and giving greater emphasis to the insights of the ethics of care and vulnerability approaches in the design and justification of social welfare programs.

Precisely how this ought to be done in practice is beyond the scope of this paper, which has focused on the theoretical justifications for taking embodiment seriously. Important questions remain concerning whose views should be incorporated into the policymaking process, how the facts about people’s embodiment ought to be solicited, and how this information should be used in the process of crafting public policy. For the purposes of this paper the important point is that embarking on this project does not require the wholesale rejection of liberal political philosophy and mainstream ethical theorising.

## Footnotes

1 Fineman, Martha Albertson. (2008) ‘The Vulnerable Subject: Anchoring Equality in the Human Condition’ Yale Journal of Law and Feminism, Vol. 20, p. 19.

2 Fineman, Martha Albertson. (2008) ‘The Vulnerable Subject: Anchoring Equality in the Human Condition’ Yale Journal of Law and Feminism, Vol. 20, p. 2.

3 Ibid. p. 18.

4 Tilman, Rachel. (2013) ‘Ethical Embodiment and Moral Reasoning: A Challenge to Peter Singer’ Hypatia, Vol. 28, No. 1, p. 20.

5 Ibid. p. 2.

6 Diprose, Rosalyn. (1994) The Bodies of Women: Ethics, Embodiment, and Sexual Difference. Routledge, London. p. 16.

7 Okin, Susan Moller. (1991) ‘John Rawls: Justice as Fairness – For Whom?’ in Shanley, Mary Lyndon and Pateman, Carole. (Eds) *Feminist Interpretations and Political Theory.* The Pennsylvania State University Press, University Park PA. p. 181; Weiss, Gail. (2018) ‘A Genealogy of Women’s (Un)Ethical Bodies’ in Fisher, Clara and Dolezal, Luna (Eds) *New Feminist Perspectives on Embodiment*, Palgrave MacMillan, Cham Switzerland. p 27; Engster, Daniel. (2015)* Justice, Care, and the Welfare State.* Oxford University Press, Oxford. p. 14; Engster, Daniel. (2007) The Heart of Justice. Oxford University Press, Oxford. pp. 8-9.

8 Okin, Susan Moller. (1991) ‘John Rawls: Justice as Fairness – For Whom?’ in Shanley, Mary Lyndon and Pateman, Carole. (Eds) *Feminist Interpretations and Political Theory.* The Pennsylvania State University Press, University Park PA. p. 181; Nussbaum, Martha. (2006) *Frontiers of Justice: Disability, Nationality, Species Membership.* Harvard University Press, Cambridge MA. p. 18; Pateman, Carole. (1988) The Sexual Contract. Polity Press, Oxford. p. 41.

9 Burkitt, Ian. (1999) *Bodies of Thought*. SAGE, London. p. 104; Mills, Sophie. (1999) ‘Owning my ‘Self’: a reconciliation of perspectives on the body’ UCL Juris Rev, Vol. 6, p. 195; Kingdom, Elizabeth. (1995) ‘Body Politics and Rights’ in Bridgeman, Jo & Millns, Susan. *Law and Body Politics: Regulating the Female Body*. Dartmouth Publishing, Aldershot. p. 4; Dolezal, Luna (2015) The Body and Shame: Phenomenology, Feminism, and the Socially Shaped Body. Lexington Books, Lanham. p. 124.

10 Kingdom, Elizabeth. (1995) ‘Body Politics and Rights’ in Bridgeman, Jo & Millns, Susan. *Law and Body Politics: Regulating the Female Body*. Dartmouth Publishing, Aldershot.

11 Diprose, Rosalyn. (1994) The Bodies of Women: Ethics, Embodiment, and Sexual Difference. Routledge, London.

12 Hamilton, Sheryl N. (2009) *Impersonations: Troubling the Person in Law and Culture.* University of Toronto Press, Toronto.

13 Sandel, Michael. (1984) *Liberalism and the Limits of Justice*. New York University Press, New York. p. 175.

14 Taylor, Charles (1985) Philosophy and the Human Sciences: Philosophical Papers 2. Cambridge University Press, Cambridge. p. 190.

15 MacIntyre, Alasdair. (2006) ‘What is a Human Body?’ in *The Tasks of Philosophy.* Cambridge University Press, Cambridge; MacIntyre, Aladair. (2007) After Virtue. University of Notre Dame Press, Notre Dame Indiana.

16 Leder, Drew. (1994) ‘A Tale of Two Bodies: The Cartesian Corpse and the Lived Body’ in Leder, Drew (Ed) *The Body in Medical Thought and Practice.* Springer.

17 Svenaeus, Fredrik. (2018) Phenomenological Bioethics: Medical Technologies, Human Suffering, and the Meaning of Being Alive. Routledge, Abingdon.

18 Toombs, S. Hay. (1987) ‘The Meaning of Illness: A Phenomenological Approach to the Patient-Physician Relationship’ *The Journal of Medicine and Philosophy,* Vol. 12, pp. 229.

19 Kittay, Eva Feder. (2011) ‘The Ethics of Care, Dependence, and Disability’ *Ration Juris,* Vol. 24, No. 1, p. 50.

20 Shildrick, Margrit. (2015) ‘‘Why Should Our Bodied End at the Skin?’: Embodiment, Boundaries, and Somatechnics’ *Hypatia,* Vol 30, No. 1, pp. 13-29.

21 Scully, Jackie Leach. (2008) *Disability Bioethics: Moral Bodies, Moral Difference*. Rowman & Littlefield Publishers, Lanham.

22 Burkitt, Ian. (1999) *Bodies of Thought*. SAGE, London.

23 Dolezal, Luna. (2015) *The Body and Shame*. Lexington Books, Lanham.

24 Heyes, Cressida. (2007) *Self-Transformations: Foucault, Ethics, and Normalized Bodies.* New York, Oxford University Press.

25 Kittay, Eva Feder. (1999) Love’s Labour: Essays on Women, Equality, and Dependency. Oxford, Oxford University Press. p. 4.

26 Fineman, Martha Albertson. (2004) The Autonomy Myth: A Theory of Dependency. New York, The New Press, p. 68.

27 Walker, Margaret Urban. (1998) Moral Understandings: A Feminist Study in Ethics. New York, Routledge. p. 20.

28 Fineman ‘Vulnerability and Inevitable Inequality’ p. 148; Burkitt, Ian. (1999) *Bodies of Thought*. SAGE, London. p. 90; Hamilton, Sheryl N. (2009) *Impersonations: Troubling the Person in Law and Culture.* University of Toronto Press, Toronto. p. 70; Naffine, Ngaire. (2004) ‘Our legal lives as men, women, and persons’ Legal Studies, Vol. 24, No. 4, p. 638.

29 Sandel, Michael. (1984) *Liberalism and the Limits of Justice*. New York University Press, New York. MacIntyre, Alasdair. (2007) After Virtue. Notre Dame University Press, Notre Dame IN.

30 Kittay, Eva Feder. (2011) ‘The Ethics of Care, Dependence, and Disability’ *Ratio Juris,* Vol. 24, No. 1, p. 50; MacIntyre, Alasdair. (1999) Dependent Rational Animals. Open Court Publishing, Chicago. p. 5; Thomas, Carol. (2007) *Sociologies of Disability and Illness.* Palgrave Macmillan, Basingstoke. p. 140; Nussbaum, Martha. (2006) Frontiers of Justice. Harvard University Press, Cambridge MA. p. 15.

31 Thomson, Rosemary Garland (1997) *Extraordinary Bodies*. Columbia University Press, NYC. p. 40.

32 Young, Iris Marion. (1980) ‘Throwing like a Girl: A Phenomenology of Feminine Comportment, Motility and Spatiality’ *Human Studies,* Vol. 3, No. 2, p. 142; Toombs, S Kay. (2002) ‘What Does it Mean to be SomeBody? Phenomenological Reflections and Ethical Quandries’ in Cherry, Mark. (Ed) Persons and Their Bodies: Rights, Responsibilities, Relationships. Kluwer Academic Publishers, New York. pp. 73-94; Carel, Havi. (2016) Phenomenology of Illness. Oxford University Press, Oxford.

33 Fineman, Martha Albertson. (2004) *The Autonomy Myth: A Theory of Dependency*. The New Press, New York. p. 50; Kittay, Eva Feder. (1999) *Love’s Labour: Essays on Women, Equality, and Dependency*. Routledge, New York; Engster, Daniel. (2019) ‘Care Ethics, Dependency, and Vulnerability’ *Ethics and Social Welfare*, Vol. 31, No. 2, p. 100.

34 Toombs, S Kay. (2002) ‘What Does it Mean to Be SomeBody? Phenomenological Reflections and Ethical Quandries’ in Cherry, Mark J. (Ed) Persons and Their Bodies: Rights, Responsibilities, Relationships’ Kluwer Academic Publishers, New York. p. 73.

35 Toombs, S Kay. (1987) ‘The Meaning of Illness: A Phenomenological Approach to the Patient-Physician Relationship’ The Journal of Medicine and Philosophy, Vol. 12, p. 229.

36 Carel, Havi. (2016) Phenomenology of Illness. Oxford University Press, Oxford. p. 68.

37 Carel, Havi. (2016) Phenomenology of Illness. Oxford University Press, Oxford. p. 71.

38 Toombs, S Kay. (2002) ‘What Does it Mean to Be SomeBody? Phenomenological Reflections and Ethical Quandries’ in Cherry, Mark J. (Ed) Persons and Their Bodies: Rights, Responsibilities, Relationships’ Kluwer Academic Publishers, New York. p. 76.

39 Toombs, S. Kay. (2019) ‘The Healing Relationship: Edmund Pellegrino’s philosophy of the physician-patient encounter’ *Theoretical Medicine and Bioethics*, Vol. 40, pp. 222.

40 Toombs, S. Kay. (2019) ‘The Healing Relationship: Edmund Pellegrino’s philosophy of the physician-patient encounter’ *Theoretical Medicine and Bioethics*, Vol. 40, pp. 222; Papadimitriou, Christina. (2008) ‘Becoming en-wheeled: the situated accomplishment of re-embodiment as a wheelchair user after spinal cord injury’ *Disability and Society*, Vol. 23, No. 7, p. 694.

41 Toombs, S Kay, (1992) ‘The Body in Multiple Sclerosis: A Patient’s Perspective’ in Leder, Drew (Ed) The Body in Medical Thought and Practice. Springer, Dordrecht. p. 130.

42 Young, Iris Marion. (1980) ‘Throwing like a Girl: A Phenomenology of Feminine Comportment, Motility and Spatiality’ *Human Studies,* Vol. 3, No. 2, p. 142.

43 Young, Iris Marion. (1980) ‘Throwing like a Girl: A Phenomenology of Feminine Comportment, Motility and Spatiality’ *Human Studies,* Vol. 3, No. 2, p. 142.

44 Young, Iris Marion. (1980) ‘Throwing like a Girl: A Phenomenology of Feminine Comportment, Motility and Spatiality’ *Human Studies,* Vol. 3, No. 2, p. 144.

45 Burkitt, Ian. (1999) Bodies of Thought: Embodiment, Identity and Modernity. SAGE, London. p. 101. See also: Jansen, Julia and Wehrle, Maren. (2018) ‘The Normal Body: Female Bodies in Changing Contexts of Normalisation and Optimisation’ in Fisher, Clara and Dolezal, Luna (Eds) *New Feminist Perspectives on Embodiment*, Palgrave MacMillan, Cham Switzerland. p 42.

46 For more information see Roberts, Joseph T F et al. (2021) ‘Prescribing unapproved medical devices? The case of DIY artificial Pancreas systems’ Medical Law International, Vol. 21, p. 46.

47 See A. Brown, ‘42 Factors That Affect Blood Glucose’, Diatribe 13 February 2018. Available at: https://www.diatribe.org/42factors (accessed 4 March 2021).

48 Roberts, Joseph T F; Moore, Victoria; and Quigley, Muireann. (2021) ‘Prescribing unapproved medical devices? The case of DIY artificial pancreas systems’ Medical Law International, Vol. 21, No. 1, p. 46; Young, Amanda et al. (2015) ‘Holistic Impact of Closed-Loop Technology on People with Type 1 Diabetes’ Journal of Diabetes Science and Technology, Vol 9, No. 4, pp. 932-933.

49 Murray, C. D. (2004) ‘An Interpretative Phenomenological Analysis of the Embodiment of Artificial Limbs’ *Disability and Rehabilitation,* Vol. 26, No. 16, p. 966; Saradjian et al ‘Experience of Men using an upper limb prosthesis’ p. 877; Murray ‘Embodiment and Prosthetics’ p. 121.

50 Murray ‘Embodiment of Artificial Limbs’ p. 966.

51 Murray ‘Embodiment of Artificial Limbs’ p. 967.

52 Murray ‘Embodiment of Artificial Limbs’ p. 967.

53 Hafner et al ‘Characterising Mobility’ p. 586.

54 Carell, Havi. (2016) *Phenomenology of Illness.* Oxford University Press, Oxford. p. 46.

55 Scully, Jackie Leach. (2018) ‘From ‘She would say that, wouldn’t she?’ to ‘Does she take sugar?’ Epistemic Injustice and Disability’ International Journal of Feminist Approaches to Bioethics, Vol. 11, No. 1, p.110.

56 Mackenzie, Catriona and Scully, Jackie Leach. (2007) ‘Moral Imagination, Disability, and Embodiment’ Journal of Applied Philosophy, Vol. 24, No. 4, p. 337; Carell, Havi. 2016) Phenomenology of Illness, Oxford University Press, Oxford. p. 10.

57 Svenaeus, Fredrik. (2018) Phenomenological Bioethics: Medical Technologies, Human Suffering, and the Meaning of Being Alive. Routledge, Abingdon. p. 9.

58 Rawls, John. (1971) *A Theory of Justice*. p. 343.

59 Karnein, Anja. (2014) ‘Putting Fairness in Its Place: Why There is a Duty to Take up the Slack’ *The Journal of Philosophy,* Vol. 111, No. 111, p. 593-607.

60 Sen, Amartya. (1992) Inequality Re-examined. Oxford University Press, Oxford. p. 20-28.

61 Scope, ‘Disability Price Tag’ Available at: https://www.scope.org.uk/campaigns/extra-costs/disability-price-tag/ (Accessed 16th June 2022).

62 Fineman, Martha Albertson. (2020) ‘Reasoning from the Body: Universal Vulnerability and Social Justice’ in Dietz, Travis and Thomson (Eds) *A Jurisprudence of the Body*. Palgrave MacMillan, Cham.p. 19.

63 Burkitt, Ian. (1999) *Bodies of Thought.* SAGE Publishing, London. p. 2.

64 Burkitt, Ian. (1999) *Bodies of Thought.* SAGE Publishing, London. p. 4, Spitzack, Carole. (1994) ‘Foucault’s Political Body in Medical Practice’ in Leder, Drew (Ed) *The Body in Medical Thought and Practice.* Springer, p. 53; Heyes, Cressida. (2007)* Self-Transformations: Foucault, Ethics, and Normalised Bodies.* Oxford University Press, New York. p. 7; Dolezal, Luna. (2015) *The Body and Shame*. Lexington Books, Lanham. p. 54.

65 Fineman, Martha Albertson. (2020) ‘Reasoning from the Body: Universal Vulnerability and Social Justice’ in Dietz, Travis and Thomson (Eds) *A Jurisprudence of the Body*. Palgrave MacMillan, Cham. p. 19; Diprose, Rosalyn. (1994)* The Bodies of Women: Ethics, Embodiment, and Sexual Difference.* Routledge, London. p. 4; Kass, Leon. (2002) *Life, Liberty and Dignity*. Encounter Books, San Francisco. p. 17.

66 Burkitt, Ian. (1999) *Bodies of Thought.* SAGE Publishing, London. p. 151; Carel, Havi. (2012) ‘Phenomenology as a Resource for Patients’ *Journal of Medicine and Philosophy*, Vol. 37, p. 105; Engster, Daniel. (2005) ‘Rethinking Care Theory: The Practice of Caring and the Obligation to Care’ Hypatia, Vol. 20, No. 3, p. 56; MacIntyre, Alasdair. (1999) *Dependent Rational Animals*. Open Court Publishing, Chicago. p. 1.

67 Engster ‘Rethinking Care Theory’ p. 60; Held, Virginia. (2014) ‘The Ethics of care as Normative Guidance: Comment on Gilligan’ *Journal of Social Philosophy*, Vol. 45, No. 1, p. 111.

68 MacIntyre, Alasdair. (1999) *Dependent Rational Animals*. Open Court Publishing, Chicago. p. 8.

69 Fineman, Martha Albertson. (2020) ‘Reasoning from the Body: Universal Vulnerability and Social Justice’ in Dietz, Travis and Thomson (Eds) *A Jurisprudence of the Body*. Palgrave MacMillan, Cham. p. 17.

70 Fineman, Martha Albertson. (2008) ‘The Vulnerable Subject: Anchoring Equality in the Human Condition’ *Yale Journal of Law and Feminism,* Vol. 20, p. 9; Fineman, Matha Albertson. ‘Equality, Autonomy, and the Vulnerable Subject in Law and Politics’ in Fineman and Grear (Eds) *Vulnerability: Reflections on a New Ethical Foundation for Law and Politics.* Routledge, London. p. 20.

71 Fineman ‘The Vulnerable Subject’ p. 8; MacIntyre ‘Dependent Rational Animals’ p. 2; Fineman, Martha Albertson. (2020) ‘Reasoning from the Body: Universal Vulnerability and Social Justice’ in Dietz, Chris; Travis, Mitchell; and Thomson, Michael. (Eds) *A Jurisprudence of the Body*. Palgrave MacMillan, Cham. p. 18; Fineman, Martha Albertson. (2013) ‘Equality, Autonomy, and the Vulnerable Subject in Law and Politics’ in Fineman, Martha Albertson and Grear, Anna (Eds) *Vulnerability: Reflections on a New Ethical Foundation for Law and Politics*. Routledge, London. p. 20; Kittay, Eva Feder. (2011) ‘The Ethics of Care, Dependence, and Disability’ *Ratio Juris*, Vol. 24, No. 1, p. 54; Engster ‘Rethinking Care’ p. 61.

72 Fineman, Martha Albertson. (2004) *The Autonomy Myth: A Theory of Dependency.* The New Press, New York. p. xiii; ; Fineman, Martha Albertson. (2008) ‘The Vulnerable Subject: Anchoring Equality in the Human Condition’ *Yale Journal of Law and Feminism*, Vol. 20, p. 5.

73 Fineman, Martha Albertson. (2017) ‘Vulnerability and Inevitable Inequality’ *Oslo Law Review*, Vol. 4, No. 3, p. 140; Fineman ‘The Vulnerable Subject’ p. 12.

74 Fineman, *The Autonomy Myth* p. 238; Fineman ‘Vulnerability and Inevitable Inequality’ p. 140; Fineman, Martha Albertson. (2019a) ‘Vulnerability and Social Justice’ Valparaiso University Law Review, Vol. 53, No. 2, p. 361.

75 Fineman, *The Autonomy Myth* p. 39.

76 Fineman, *The Autonomy Myth* p. 244.

77 Fineman, *The Autonomy Myth*. p. 251.

78 Fineman, *The Autonomy Myth* p. 285.

79 Fineman, *The Autonomy Myth* p. 285; Fineman, Martha Albertson. (2019b) ‘Vulnerability in Law and Bioethics’ Journal of Health Care for the Poor and Underserved, Vol. 30, No. 4, p. 57; Fineman ‘Vulnerability and Social Justice’p. 363.

80 Fineman, *The Autonomy Myth* p. 282; Fineman, Martha Albertson. (2013) ‘Equality, Autonomy, and the Vulnerable Subject in Law and Politics’ in Fineman, Martha Albertson and Grear, Anna. (Eds) Vulnerability: Reflections on a New Ethical Foundation for Law and Politics. Routledge, London. p. 13; Fineman ‘The Vulnerable Subject’ p. 1.

81 Engster, Daniel. (2005) ‘Rethinking Care Theory: The Practice of Caring and the Obligation to Care’ *Hypatia*, Vol. 20, No. 3, p. 55.

82 Engster, Daniel. (2005) ‘Rethinking Care Theory: The Practice of Caring and the Obligation to Care’ *Hypatia*, Vol. 20, No. 3, p. 55.

83 Kittay ‘The Ethics of Care, Dependence, and Disability’ p. 53; Gilligan, Carol. (2014) ‘Moral Injury and the Ethic of Care: Reframing the Conversation about Differences’ *Journal of Social Philosophy*, Vol. 45, No. 1, pp. 89; Diprose, Rosalyn. (1994) *The Bodies of Women: Ethics, Embodiment, and Sexual Difference*. Routledge, London. p. 9.

84 Held ‘The Ethics of Care’ p. 109.

85 Held ‘The Ethics of Care’ p. 109.

86 Kittay ‘The Ethics of Care, Dependence, and Disability’ p. 53; Gilligan ‘Moral Injury and the Ethic of Care’ p. 89.

87 Held ‘The Ethics of Care’ p. 109.

88 Kittay ‘The Ethics of Care, Dependence, and Disability’ p. 53.

89 Engster ‘Care Ethics, Dependency, and Vulnerability’ p. 105.

90 Engster ‘Rethinking Care Theory’ p. 63.

91 Held ‘The Ethics of Care’ p. 109; Engster ‘Care Ethics, Dependence and Vulnerability’.

92 Stephanie Collins (2015) *The Core of Care Ethics.* Palgrave Macmillan, Basingstoke. p. 6; Engster, Daniel. (2015)* Justice, Care, and the Welfare State.* Oxford University Press, Oxford. p. 4.

93 Kittay, Eva Feder. (2011) ‘The Ethics of Care, Dependence, and Disability’ *Ratio Juris,* Vol. 24, No. 1, p. 54.

94 Kittay, Eva Feder. (2011) ‘The Ethics of Care, Dependence, and Disability’ *Ratio Juris,* Vol. 24, No. 1, p. 56; Engster, Daniel. (2007) The Heart of Justice. Oxford University Press, Oxford. p. 68.

95 Engster The Heart of Justice pp. 80-90, 141-151; Engster Justice, Care and the Welfare State pp. 95, 187-196.

96 Dancy, Jonathan. (2017) ‘Moral Particularism’ Stanford Encyclopedia of Philosophy. Available at: https://plato.stanford.edu/archives/win2017/entries/moral-particularism/; Wisnewski, J. Jeremy. (2015) ‘Perceiving Sympathetically: Moral Perception, Embodiment, and Medical Ethics’ J Med Humanities, Vol. 36, p. 317.

97 Leder, Drew. (2002) ‘Whose Body? What Body? The Metaphysics of Organ Transplantation’ in Cherry, Mark J. (Ed) *Persons and Their Bodies: Rights, Responsibilities, Relationships.* Kluwer Academic Publishers, New York. p. 234; Gatens, Moira. (1996) *Imaginary Bodies: Ethics, Power, and Corporeality*. Routledge, London**.** p. 56; Diprose, Rosalyn. (1994) The Bodies of Women: Ethics, Embodiment, and Sexual Difference. Routledge, London. p. 17; Kass, Leon. (2002) Life, Liberty and Dignity. Encounter Books, San Francisco. p. 60; Held, Virginia (2006) *The Ethics of Care: Personal, Political, and Global.* Oxford, Oxford University Press. p. 11, p. 58; Noddings, Nel. (2002) *Starting at Home: Caring and Social Policy*. University of Carolina Press, Los Angeles. p. 30; Tillman, Rachel. (203) ‘Ethical Embodiment and Moral Reasoning: a Challenge to Peter Singer’ Hypatia, Vol. 28, No. 1, p. 20; Wisnewski, J. Jeremy. (2015) ‘Perceiving Sympathetically: Moral Perception, Embodiment, and Medical Ethics’ J Med Humanities, Vol. 36, p. 317.

98 Crisp, Roger. (2000) ‘Particularising Particularism’ in Hooker, Brad and Little, Margaret Olivia (eds) *Moral Particularism*, Oxford University Press, Oxford. p. 23.

99 Dancy, Jonathan. (2017) ‘Moral Particularism’ Stanford Encyclopedia of Philosophy. Available at: https://plato.stanford.edu/archives/win2017/entries/moral-particularism/.

100 Kass, Leon. (2002) Life, Liberty and Dignity. Encounter Books, San Francisco. p. 63; Diprose, Rosalyn. (1994) *The Bodies of Women: Ethics, Embodiment, and Sexual Difference*. Routledge, London. p. 17.

101 Vayrynen, Pekka. (2008) ‘Usable Moral Principles’ in Lance, Mark Norris; Potrc, Matjaz; and Strahovnik, Vojko (Eds) *Challenging Moral Particularism.* Routledge, London. p. 75.

102 Wisnewski, J. Jeremy. (2015) ‘Perceiving Sympathetically: Moral Perception, Embodiment, and Medical Ethics’ *Journal of Medical Humanities*, Vol. 36, p. 319.

103 Raz, Joseph. (2000) ‘The Truth in Particularism’ in Hooker, Brad and Little, Margaret Olivia (eds) *Moral Particularism*, Oxford University Press, Oxford. p. 58.

104 Toulmin, Stephen. (1981) ‘The Tyranny of Principles’ *the Hastings Centre Report,* Vol. 11, No. 6, p. 39.

105 Hooker, Brad. (2000) ‘Moral Particularism: Wrong and Bad’ in Hooker, Brad and Little, Margaret Olivia (2000) *Moral Particularism*. Oxford University Press, Oxford. p. 5.

106 Hooker, Brad. (2000) ‘Moral Particularism: Wrong and Bad’ in Hooker, Brad and Little, Margaret Olivia (2000) *Moral Particularism*. Oxford University Press, Oxford. p. 6.

107 Little, Margaret Olivia. (2001) ‘On Knowing the ‘Why’: Particularism and Moral Theory’ *Hastings Centre Report*, Vol. 31, No. 4, p. 34.

108 Ridge, Michael. (2016) ‘Moral Particularism and Moral Generalism’ *Stanford Encyclopaedia of Philosophy*. Edward N. Zalta (ed.), URL: https://plato.stanford.edu/archives/win2020/entries/moral-particularism-generalism/; Dancy, Jonathan. (2017) ‘Moral Particularism’ *The Stanford Encyclopedia of Philosophy*, Edward N. Zalta (ed.), URL = <https://plato.stanford.edu/archives/win2017/entries/moral-particularism/>.

109 Little, Margaret Olivia. (2001) ‘On Knowing the ‘Why’: Particularism and Moral Theory’ *Hastings Centre Report*, Vol. 31, No. 4, p. 34.

110 Dancy, Jonathan. (2017) ‘Moral Particularism’ *The Stanford Encyclopedia of Philosophy*, Edward N. Zalta (ed.), URL = <https://plato.stanford.edu/archives/win2017/entries/moral-particularism/>.

111 Kuczewski, Mark. (1998) ‘Casuistry and Principlism: The convergence of Method in Biomedical Ethics’, *Theoretical Medicine and Bioethics,* vol. 10, p. 511; Little, Margaret Olivia. (2001) ‘On Knowing the ‘Why’: Particularism and Moral Theory’ *Hastings Centre Report*, Vol. 31, No. 4, p. 32.

112 DeGrazia, David and Millum, Joseph. (2021) *A Theory of Bioethics*. Cambridge University Press, Cambridge. p. 17.

113 Hooker, Brad. (2000) ‘Moral Particularism: Wrong and Bad’ in Hooker, Brad and Little, Margaret Olivia (2000) *Moral Particularism*. Oxford University Press, Oxford. p. 7.

114 Ridge, Michael. (2016) ‘Moral Particularism and Moral Generalism’ *Stanford Encyclopaedia of Philosophy*. Edward N. Zalta (ed.), URL: https://plato.stanford.edu/archives/win2020/entries/moral-particularism-generalism/.

115 Nussbaum, Martha. (2000) ‘Why Practice needs Ethical Theory: Particularism, Principle, and Bad Behaviour’ in Hooker, Brad and Little, Margaret Olivia (2000) *Moral Particularism*. Oxford University Press, Oxford. p. 241.

116 Dancy, Jonathan. (2017) ‘Moral Particularism’ *The Stanford Encyclopedia of Philosophy*, Edward N. Zalta (ed.), URL = <https://plato.stanford.edu/archives/win2017/entries/moral-particularism/>.

117 Hooker, Brad. (2000) ‘Moral Particularism: Wrong and Bad’ in Hooker, Brad and Little, Margaret Olivia (2000) *Moral Particularism*. Oxford University Press, Oxford. p. 6.

118 DeGrazia, David and Millum, Joseph. (2021) *A Theory of Bioethics*. Cambridge University Press, Cambridge. p. 23.

119 Vayrynen, Pekka. (2008) ‘Usable Moral Principles’ in Lance, Mark Norris; Potrc, Matjaz; and Strahovnik, Vojko (Eds) *Challenging Moral Particularism.* Routledge, London. p. 93.

120 DeGrazia, David and Millum, Joseph. (2021) *A Theory of Bioethics*. Cambridge University Press, Cambridge. p. 13.

121 Cummins, Robert. (1998) ‘Reflection on Reflective Equilibrium’ in DePaul, Michael R and Ramsey, William (Eds) Rethinking Intuition. Lantham MD, Rowman & Littlefield. p. 113.

122 Daniels, Norman. (1979) ‘Wide Reflective Equilibrium and Theory Acceptance in Ethics’ *The Journal of Philosophy,* Vol. 76, No. 5, p. 259.

123 Vayrynen, Pekka. (2008) ‘Usable Moral Principles’ in Lance, Mark Norris; Potrc, Matjaz; and Strahovnik, Vojko (Eds) *Challenging Moral Particularism.* Routledge, London. p. 87.

124 Thank you to an anonymous reviewer who pointed this objection out to me.

125 Okin, Susan Moller. (1989) ‘Reason and Feeling in Thinking about Justice’ Ethics, Vol. 99, No. 2, p. 229; Darwall, Stephen. (1998) *Philosophical Ethics*. Routledge, New York. p.228.

126 Collins, *The Core of Care Ethics*. p. 5; Kittay, Eva Feder. (1999) *Love’s Labour.* Routledge, New York. p. 19; Tronto, Joan. (1993) *Moral Boundaries: A Political Argument for an Ethic of Care.* Routledge, New York. p. 152.

127 Engster ‘Care Ethics, Dependence, and Vulnerability’ p. 107.

128 Collins The Core of Care Ethics, p. 99.

129 Collins *The Core of Care Ethics* p. 32; Noddings, Nel. (2013/1986) *Caring: A Relational Approach to Ethics and Moral Education*. University of California Press, Berkley. pp. 1, 5, 7, 25.

130 Mills, Catherine. (2007) ‘Normative Violence, Vulnerability, and Responsibility’ Differences, Vol. 18, p. 142.

131 Engster ‘Rethinking Care Theory’ p. 64; Daniel Engster (2007) The Heart of Justice. Oxford University Press, Oxford. p. 6.

132 Engster, Daniel. (2015) *Justice, Care, and the Welfare State*. Oxford University Press, Oxford. p. 22.

133 Engster, The Heart of Justice, p. 51.

134 Dancy, Jonathan. (2017) ‘Moral Particularism’ *The Stanford Encyclopedia of Philosophy*, Edward N. Zalta (ed.), URL = <https://plato.stanford.edu/archives/win2017/entries/moral-particularism/>.

135 Engster Justice, Care and the Welfare State p. 104-114.

136 Collins *The Core of Care Ethics* p. 1.

137 Collins *The Core of Care Ethics* p. 1.

138 Collins, The Core of Care Ethics p. 108.

139 Virginia Held (2006) *The Ethics of Care: Personal, Political, Global.* Oxford University Press, Oxford. p. 63; Noddings, Nel. (1984/2013) *Caring: A Relational Approach to Ethics and Moral Education*. University of California Press, Berkley. p. 8; Gilligan, Carol. (1982) In A Different Voice: Psychological Theory and Women’s Development. Harvard University Press, Cambridge MA. p. 69, 90; Slote, Michael. (2007) *The Ethics of Care and Empathy.* Routledge, Abingdon. p. 1.

140 Collins *The Core of* Care Ethics p. 6; Engster, *Justice, Care and the Welfare State* p. 24.

141 Collins *The Core of* Care Ethics p. 6, Daniel Engster (2007) *The Heart of Justice.* Oxford University Press, Oxford. p. 6; Virginia Held (2006) *The Ethics of Care: Personal, Political, Global.* Oxford University Press, Oxford. p. 16.

142 Okin ‘Reason and Feeling’ p. 238.

143 Kittay, Eva Feder. (2015) ‘Centering Justice on Dependence and Recovering Freedom’ Hypatia, Vol. 30, No. 1, p. 288; Held *The Ethics of Care* p. 69.

144 Engster, Justice, Care, and the Welfare State p. 82.

145 Engster Justice, Care, and the Welfare State p. 17.

146 Held The Ethics of Care p. 40.

147 Engster, The Heart of Justice. p. 93.

148 Held *The Ethics of Care* p. 17.
